# On the Applications of Discontinuous Bessel Integrals to Chronoamperometry

**DOI:** 10.6028/jres.100.006

**Published:** 1995

**Authors:** William T. Yap, Richard A. Durst

**Affiliations:** National Institute of Standards and Technology, Gaithersburg, MD 20899-0001; Cornell University, Geneva, NY 14456

**Keywords:** asymptotic expansion, boundary value problem, chronoamperometry, discontinuous Bessel integral, Laplace transform

## Abstract

Discontinuous Bessel integrals are applied to a boundary value problem related to chronoamperometry, with zero concentration at the disk satisfied on the average and the zero flux at the shroud satisfied approximately only. Current functions are derived, series expansion at long time and asymptotic expansion at short times are given. Plots of numerical calculations of current functions are presented.

## 1. Introduction

**Fig. 1:**
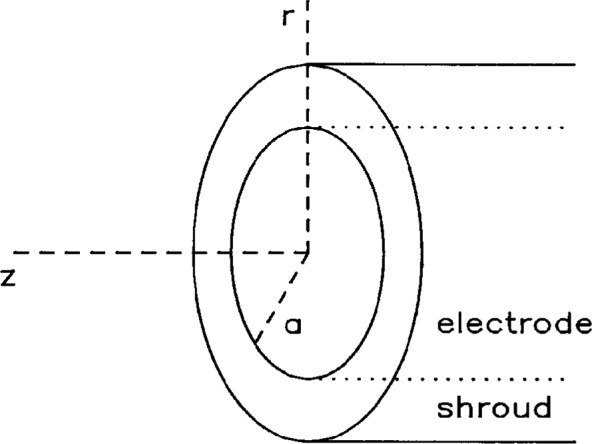
Shrouded electrode.

Chronoamperometry, where a current versus time curve is measured after a step potential is applied to the electrode, is one of the important techniques in electro-analytical chemistry. The analysis of chronoamperometry at a shrouded circular disk electrode (see Fig. 1) involves solving boundary value problems with boundary and initial conditions that give rise to discontinuities in concentration and flux distribution at the disk/shroud boundary. Discontinuous integrals of Bessel functions, which are solutions to the diffusion equations, have this property; some of them have been used in heat conduction in cylindrical geometry [1]. They have also been nicely applied in treating steady state problems in electroanalytical chemistry [2,3]. However applications of these integrals in transient processes, such as chronoamperometry, at the shrouded disk electrode become more difficult and may involve current functions in the Laplace domain that can not be easily inverted analytically [4,5]. We shall report in this paper some of the mathematical results we obtained in applying a class of these discontinuous Bessel integrals to a boundary value problem related to that of chronoamperometry and compare our results with some of the results in chronoamperometry.

## 2. Concentration Distribution

Consider a disk electrode with radius *a*, and let the origin of a cylindrical coordinate system be placed at the center of the disk with *z*-axis perpendicular to the disk. Also consider a reaction at the electrode, Ox+*n*e→Red, and let the concentration of Ox, *C*(*r*,*z*,*t*), be *C*^0^ throughout the solution initially. At time *t*=0, let the potential be stepped to a large negative value such that the concentration of Ox at the electrode is zero. Then *C*(*r*,*z*,*t*) satisfies the diffusion equation,

$$\partial C(r,z,t)/\partial t=D[\partial^{2}/\partial r^{2}+r^{-1}\partial /\partial r+\partial^{2}/\partial z^{2}]C(r,z,t)$$

with the following initial and boundary conditions:

$$\begin{array}{l} \begin{array}{ll} C(r,z,0)=C^{0} & at\;t=0\\ C(r,0,t)=0 & for\;0<r<a,\;t>0 \end{array}\\ \underset{r\to \infty }{\lim}C(r,z,t)\to C^{0}\\ \underset{z\to \infty }{\lim}C(r,z,t)\to C^{0} \end{array}$$

and *D* denotes the diffusion coefficient. For the commonly used shrouded electrode there is an additional boundary condition that the flux into the shroud is zero. The mixed boundary conditions of zero flux at the shroud and zero concentration at the disk make a more difficult mathematical problem. Various techniques have been applied to this boundary value problem with various degree of rigor [6–9]. We shall consider the limiting case where the thickness of the shroud approaches zero and ignore the zero flux condition for the moment.

Let us define the following dimensionless variables: *x*=*r*/*a*, *y*=*z*/*a*, *u*=*Dt*/*a*^2^, and *c*(*x*,*y*,*u*)= *C*(*r*,*z*,*t*)/*C*^0^−1; then the above diffusion equation and the boundary conditions become,

$$\partial c(x,y,u)/\partial u=(\partial^{2}/\partial x^{2}+x^{-1}\partial /\partial x+\partial^{2}/\partial y^{2})c(x,y,u)$$ (1)

c(x,y,0)=0 (2)

c(x,0,u)=−1for0<x<1 (3)

$$\underset{x\to \infty }{\lim}c(x,y,u)=\underset{y\to \infty }{\lim}c(x,y,u)=0$$ (4)

Taking Laplace transforms of Eq. (1) with the initial condition Eq. (2), we obtain,

$$(\partial^{2}/\partial x^{2}+x^{-1}\partial /\partial x+\partial^{2}/\partial y^{2}-p)\bar{c}(x,y,p)=0$$ (5)

where *p* denotes the Laplace transform variable. Equation (5) is solved by the method of separation of variables by letting $\bar{c}(x,y,p)=X(x,p)Y(y,p)$. Substituting this into Eq. (5) the following two ordinary differential equations are obtained

$$(d^{2}/dx^{2}+x^{-1}d/dx+\xi^{2})X(x,p)=0$$ (6)

$$(d^{2}/dy^{2}-\lambda^{2})Y(y,p)=0$$ (7)

where *ξ*^2^ is the separation parameter and *λ*^2^= *ξ*^2^+*p*. The solutions to Eqs. (6) and (7), with the boundary conditions of Eq. (4), are the Bessel functions *J*_0_(*ξx*) and the exponentials exp(−*λy*), for *X*(*x*,*p*) and *Y*(*y*,*p*) respectively; we denote the set of product solutions to Eq. (5) by *g*(*ξ*)*J*_0_(*ξx*)exp(−*λy*), and try to satisfy the discontinuous boundary condition Eq. (3) by combinations of product solutions with various *ξ*. To satisfy Eq. (3), we try to make use of some of the discontinuous integrals of Bessel functions [10] such that ∫*g*(*ξ*)*J*_0_(*ξx*)d*ξ*=−1/*p* for 0<*x*<1. One of the well known discontinuous Bessel integral with *g*(*ξ*)=*k* sin*ξ*/*ξ* will give a constant for 0<*x*<1, but it does not give a value of 0 for all *x*>1 at *t*=0, Eq. (2). We shall try a class of discontinuous Bessel integrals of Weber with *g*(*ξ*)=*kJ_μ_* (*ξ*)/*ξ^μ^*^−1^, writing $\bar{c}(x,y,p)$ as an integral, i.e., $\bar{c}(x,y,p)=k\int_{0}^{\infty }\xi^{1-\mu }J_{\mu }(\xi )J_{0}(\xi x)\exp(-\lambda y)d\xi$. At *y*=0,

$$\begin{array}{l} \bar{c}(x,0,p)=k\int_{0}^{\infty }\xi^{1-\mu }J_{\mu }(\xi )J_{0}(\xi x)d\xi \\ \begin{array}{ll} \;=k{\left((1-x^{2})/2\right)}^{\mu -1}/\Gamma (\mu ) & \text{for}\;0<x<1\\ \;=0 & \text{for}\;1<x<\infty \end{array} \end{array}$$ (8)

For *μ*=1 and *k*=−1/*p*, the boundary condition at the disk, Eq. (3), is satisfied and also *c*=0 for *x*>1. This is the only member of this group of functions that satisfies the condition that *c*(*x*,0,*t*) is uniformly equal to −1 at all *x* less than 1. However if instead we only require that Eq. (3) be satisfied on the average, i.e., $\langle c(x,0,u)\rangle =\int_{0}^{1}c(x,0,u)xdx/\int_{0}^{1}xdx=-1$, then the other members of this group satisfy this condition with *k*=−2*^μ^*^−1^ *Γ* (*μ*+1)/*p*. We shall proceed with this and take the inverse transform to obtain,

$$\begin{array}{c} c(x,y,u)=L^{-1}(-2^{\mu -1}\Gamma (\mu +1)/p)\\ \int_{0}^{\infty }\xi^{1-\mu }J_{\mu }(\xi )J_{0}(\xi x)\exp[-y{\left(p+\xi^{2}\right)}^{1/2}d\xi \\ =-\frac{\Gamma (\mu +1)}{2^{2-\mu }}\int_{0}^{\infty }\frac{J_{\mu }(\xi )J_{0}(\xi x)}{\xi^{\mu -1}}. \end{array}$$

$$\begin{array}{c} \left[e^{y\xi }erfc(y/2\sqrt{u}+\sqrt{u}\xi \right)\\ +e^{-y\xi }erfc(y/2\sqrt{u}-\sqrt{u}\xi )]d\xi \end{array}$$ (9)

## 3. Flux Distribution

The flux at *y*=0 is given by

$$\begin{array}{c} {\left(\frac{\partial c}{\partial y}\right)}_{y=0}=\frac{\Gamma (\mu +1)}{2^{1-\mu }}\{\int_{0}^{\infty }\frac{J_{\mu }(\xi )J_{0}(\xi x)erf(\xi \sqrt{u})}{\xi^{\mu -2}}d\xi \\ +\frac{1}{\sqrt{\pi u}}\int_{0}^{\infty }\frac{J_{\mu }(\xi )J_{0}(\xi x)e^{-u\xi^{2}}}{\xi^{\mu -1}}d\xi \}. \end{array}$$ (10)

**Fig. 2:**
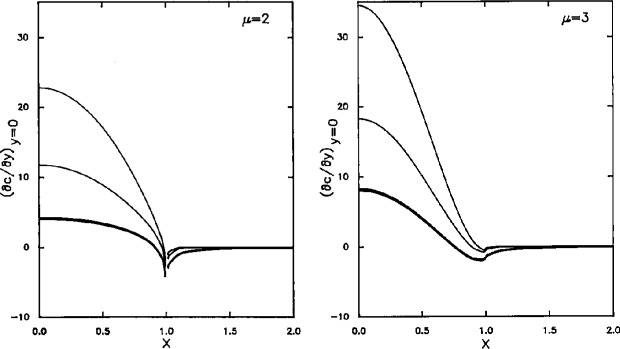
Fluxes as functions of radial distance and time. From top to bottom: $\sqrt{u}=0.05$, 0.1, 0.5, 1.0.

For ease of calculations, the first integral is written as

$$\begin{array}{c} \int_{0}^{\infty }\frac{J_{\mu }(\xi )J_{0}(\xi x)erf(\sqrt{u}\xi )}{\xi^{\mu -2}}d\xi =\int_{0}^{\infty }\frac{J_{\mu }(\xi )J_{0}(\xi x)}{\xi^{\mu -2}}d\xi \\ -\int_{0}^{\infty }\frac{J_{\mu }(\xi )J_{0}(\xi x)erfc(\sqrt{u}\xi )}{\xi^{\mu -2}}d\xi \\ =\frac{2^{2-\mu }\Gamma (3/2)}{\Gamma (3/2-\mu )}F(3/2,3/2-\mu ;1;x^{2})\\ -\int_{0}^{\infty }\frac{J_{\mu }(\xi )J_{0}(\xi x)erfc(\sqrt{u}\xi )}{\xi^{\mu -2}}d\xi \;x<1\\ =-\frac{2^{-\mu }}{x^{3}\Gamma (\mu +1)}F(3/2,3/2;\mu +1;x^{-2})\\ -\int_{0}^{\infty }\frac{J_{\mu }(\xi )J_{0}(\xi x)erfc(\sqrt{u}\xi )}{\xi^{\mu -2}}d\xi \;x>1 \end{array}$$ (11)

for *μ*>1, where *F*(*a*,*b*;*c*;*z*) denotes the Gauss hypergeometric series [11]. Figure 2 illustrates (∂*c*/∂*y*) at *y*=0 as functions of *x* and *u*, for *μ*=2 and 3. For *x*>1, (∂*c*/∂*y*)*_y_*_=0_ is nearly zero, this is particularly true for small *u* and for *x* away from the neighborhood of *x*=1. And as *u* approaches 0, the first integral in Eq. (10) approaches 0 and the second integral tends to $\int_{0}^{\infty }\xi^{1-\mu }J_{\mu }(\xi )J_{0}(\xi x)d\xi$ [12] which is identically 0 for *x*>1, Eq. (8); and the second term in Eq. (10) numerically becomes almost 0 for *x*>1 as *u*→0. Thus the concentration distribution given by Eq. (9) approximately satisfies the condition of zero flux at *x*>1, i.e., at the shroud. There is a singularity in (∂*c*/∂*y*)*_y_*_=0_ at the point *x*=1 for the case of *μ*=2, because *F*(*a*,*b*,*c*;1) is divergent for *μ*⩽2; nevertheless, the current, which is the integral of the flux over the disk area, is regular for *μ*=2.

## 4. Current as Function of Time

The current is given by *i*(*t*)=*nFD*∫(∂*C*/∂*z*)*_z_*_=0_2π*r*d*r*, integrating over the disk, where *F* denotes the Faraday constant. Defining a dimensionless current function *ϕ* (*u*)=*i*(*t*)/*2*π*nFDC*^0^*a* we get

$$\begin{array}{c} \phi (u)=\int_{0}^{1}{\left(\partial c/\partial y\right)}_{y=0}xdx\\ =\int_{0}^{1}\frac{\Gamma (\mu +1)}{2^{1-\mu }}[\int_{0}^{\infty }\frac{J_{\mu }(\xi )J_{0}(\xi x)erf(\xi \sqrt{u})}{\xi^{\mu -2}}d\xi \\ +\frac{1}{\sqrt{\pi u}}\int_{0}^{\infty }\frac{J_{\mu }(\xi )J_{0}(\xi x)e^{-\mu \xi^{2}}}{\xi^{\mu -1}}d\xi ]xdx\\ =\frac{\Gamma (\mu +1)}{2^{1-\mu }}[\int_{0}^{\infty }\frac{J_{\mu }(\xi )J_{1}(\xi )erf(\xi \sqrt{u})}{\xi^{\mu -1}}d\xi \\ +\frac{1}{\sqrt{\pi u}}\int_{0}^{\infty }\frac{J_{\mu }(\xi )J_{1}(\xi )e^{-\mu \xi^{2}}}{\xi^{\mu }}d\xi ] \end{array}$$ (12)

When *u*→0, i.e., when *t*<<*a*^2^/*D*, the second term on the right hand side of Eq. (12) dominates and

$$\underset{u\to 0}{\lim}\sqrt{u}\phi (u)=\frac{\Gamma (\mu +1)}{2^{1-\mu }\sqrt{\pi }}\int_{0}^{\infty }\frac{J_{\mu }(\xi )J_{1}(\xi )}{\xi^{\mu }}d\xi =\frac{1}{2\sqrt{\pi }}$$

or, in terms of the physical variables,

$$\begin{array}{l} \;\lim\;i(t)=\frac{nFC^{0}\sqrt{\pi D}a^{2}}{\sqrt{t}}\\ \frac{Dt}{a^{2}}\to 0 \end{array}$$

which is the Cottrell equation.

### Series expansion for large u

The last integral in Eq. (12) can be evaluated for large *u* from the following relation [13],

$$\begin{array}{c} \varphi_{2}(u)=\int_{0}^{\infty }\frac{J_{\mu }(\xi )J_{1}(\xi )e^{-u\xi^{2}}}{\xi^{\mu }}d\xi \\ =-2^{-\mu }\sum_{m=0}^{\infty }\frac{\Gamma (\mu +2m+2){\left(-4u\right)}^{-(m+1)}}{\Gamma (\mu +m+2)\Gamma (\mu +m+1)\Gamma (m+2)} \end{array}$$

and the first integral on the right hand side of Eq. (12) from

$$\begin{array}{c} \varphi_{1}(u)=\int_{0}^{\infty }\frac{J_{\mu }(\xi )J_{1}(\xi )erf(\xi \sqrt{u})}{\xi^{\mu -1}}d\xi \\ =\frac{2^{-\mu }\Gamma (\mu -1)}{\Gamma (\mu +1/2)\Gamma (\mu -1/2)}-\frac{1}{\sqrt{\pi }}\int_{\infty }^{u}\frac{1}{\sqrt{\alpha }}(d\varphi_{2}(\alpha )/d\alpha )d\alpha . \end{array}$$

Then the current, Eq. (12), is evaluated by

$$\begin{array}{c} \phi (u)=\frac{\Gamma (\mu +1)}{2}[\frac{\Gamma (\mu -1)}{\Gamma (\mu +\frac{1}{2})\Gamma (\mu -\frac{1}{2})}\\ -\frac{1}{\sqrt{\pi u}}\sum_{m=1}^{\infty }\frac{\Gamma (\mu +2m){\left(-4u\right)}^{-m}}{(2m+1)\Gamma (\mu +m+1)\Gamma (\mu +m)m!}] \end{array}$$

which is good for large value of *u*, i.e., for long time.

### Asymptotic expansion at short time

We now seek an asymptotic expansion at small *u* for *f ϕ* (*u*). Let us change the integration variable in *φ*_2_(*u*), the last integral in Eq. (12), from *ξ* to *τ*= *ξ*^2^,

$$\varphi_{2}(u)=1/2\int_{0}^{\infty }\frac{J_{\mu }(\sqrt{\tau })J_{1}(\sqrt{\tau })}{\tau^{(\mu +1)/2}}e^{-\mu \tau }d\tau .$$

Thus *φ*_2_(*u*) may be regarded as the Laplace transform of the function

$$f(\tau )=\frac{1}{2\tau^{(\mu +1)/2}}J_{\mu }(\sqrt{\tau })J_{0}(\sqrt{\tau }).$$

The Mellin transform of *f*(*τ*) is [14],

$$\begin{array}{c} F(z)=\int_{0}^{\infty }\tau^{z-1}f(\tau )d\tau \\ =\frac{{\left(1/2\right)}^{-2z+\mu +2}\Gamma (\mu -2z+2)\Gamma (z)}{\Gamma (\mu -z+1)\Gamma (\mu -z+2)\Gamma (2-z)} \end{array}$$

which is absolutely convergent for 0<*Re z*< (*μ*+3)/2. We therefore apply the results of Handelsman and Lew for the asymptotic expansion of Laplace transform near the origin [15,16] to obtain

$$\begin{array}{l} \varphi_{2}(u)=(Lf)(u)={\left(2\pi i\right)}^{-1}\int_{c-i\infty }^{c+i\infty }F(z)\Gamma (1-z)u^{z-1}dz\\ \;0<c<1\\ =\frac{2^{-\mu }}{\Gamma (\mu +1)}+\sum_{m=0}\frac{{\left(-2\right)}^{m}\Gamma (\frac{\mu +m+2}{2})u^{(\mu +m-1)/2}}{m!\Gamma (\frac{\mu -m+2}{2})\Gamma (\frac{\mu -m}{2})(\mu +m)} \end{array}$$

and the first integral, *φ*_1_(*u*), is derived from

$$\varphi_{1}(u)=-\frac{1}{\sqrt{\pi }}\int_{0}^{u}\frac{1}{\sqrt{\alpha }}(d\varphi_{2}(\alpha )/d\alpha )d\alpha .$$

Therefore, at small *u* the current, Eq. (12), is given by,

$$\begin{array}{l} \phi (u)=\frac{1}{2\sqrt{\pi }}[\frac{1}{\sqrt{u}}-2^{\mu }\Gamma (\mu +1)\\ \sum_{m=0}\frac{{\left(-2\right)}^{m}\Gamma (\frac{\mu +m+2}{2})u^{(\mu +m-1)/2}}{m!\Gamma (\frac{\mu -m+2}{2})\Gamma (\frac{\mu -m}{2})(\mu +m+1)(\mu +m)}]. \end{array}$$

**Fig. 3:**
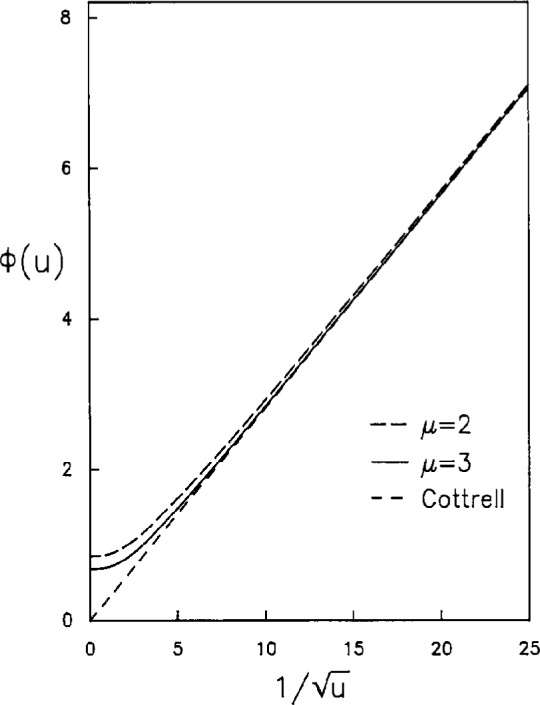
*ϕ*(*u*) as functions of $1/\sqrt{u}$.

**Fig. 4:**
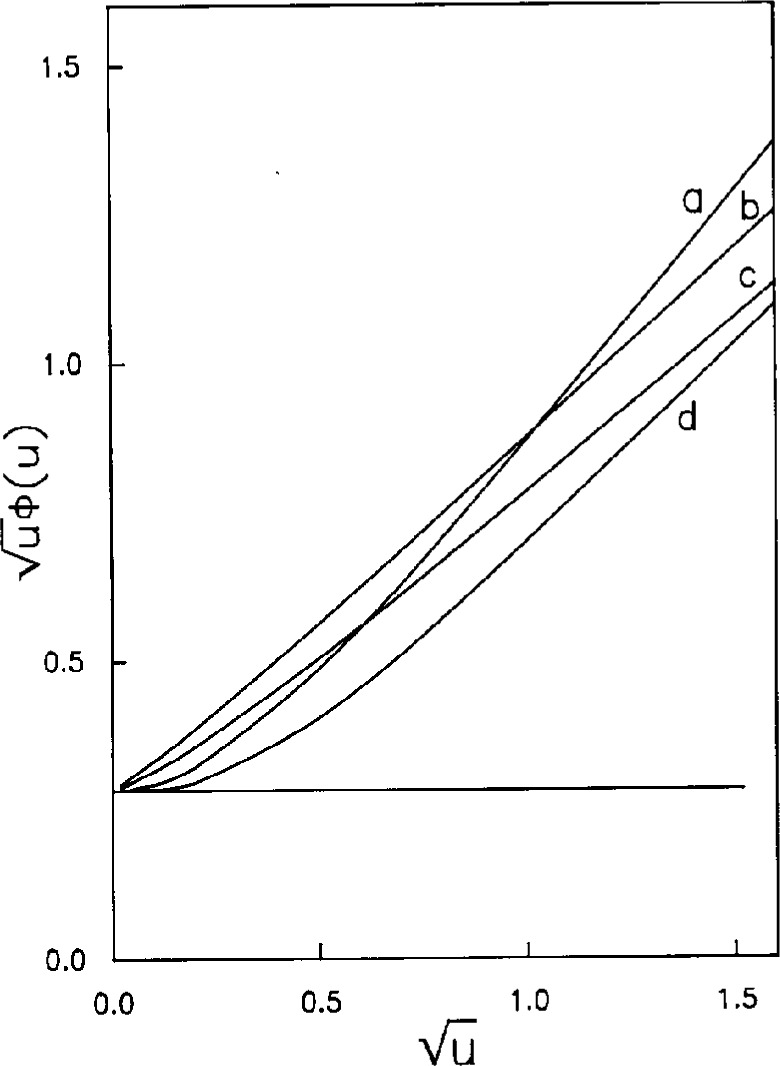
$\sqrt{u}\phi (u)$ as functions of $\sqrt{u}$. (a) *μ*=2, (b) digital simulation, (c) Ref. [2], (d) *μ*=3. Horizontal line is Cottrell one dimensional diffusion.

Several *ϕ*(*u*) versus $1/\sqrt{u}$ plots are shown in Fig. 3, including the Cottrell equation for the one dimensional diffusion, which is the straight line through the origin. The intercepts of the curves at the vertical axis show the steady state current, *ϕ*(*u*→∞), for *μ*=2 and 3, and they have the values 0.848 and 0.678, respectively; while from numerical simulation of the chronoamperometry at inlaid disk electrode [17,9], *ϕ*(*u*→∞)=0.637 which corresponds to a steady state current of *i*(*t*→∞)=4*nFDC*^0^*a*. As *μ* increases $\phi (u\to \infty )=\Gamma (\mu +1)\Gamma (\mu -1)/2\Gamma (\mu +\frac{1}{2})\Gamma (\mu -\frac{1}{2})$ decreases monotonically to 0, i.e., *ϕ*(*u*) approaches Cottrell behavior as *μ* becomes large. In Fig. 4, $\sqrt{u}\phi (u)$ for *μ*=2 and 3 are plotted versus $\sqrt{\mu }$ to magnify results at small *u*. The results from numerical simulations for shrouded disk electrode are also included, curve b. Discontinue Bessel integral was used differently in chronoamperometry by Fleischman and Pons [5], their result is also shown in Fig. 4, curve c.

## References

[1] Carslaw HS, Jaeger JC (1959). Chapter 8 in Conduction of Heat in Solids.

[2] Fleischmann M, Pons S (1987). J Electroanal Chem.

[3] Bond AM, Oldham KB, Zoski CG (1988). J Electroanal Chem.

[4] Fleischmann M, Pons S (1988). J Electroanal Chem.

[5] Fleischmann M, Pons S (1988). J Electroanal Chem.

[6] Soos ZG, Lingane PJ (1964). J Phys Chem.

[7] Oldham KB (1981). J Electroanal Chem.

[8] Oaki K, Osteryoung J (1981). J Electroanal Chem.

[9] Shoup D, Szabo A (1982). J Electroanal Chem.

[10] Watson GN (1944). Chapter 13 in Bessel Functions.

[11] Abramowitz M, Stegnum LA (1964). Handbook of Mathematical Functions.

[12] Olver FWJ (1974). SIAM J Math Anal.

[13] Erdelyi A, Magnus W, Oberhettinger F, Tricomi FC (1954). Tables of Integral Transforms.

[14] Oberhettinger F (1974). Tables of Mellin Transforms.

[15] Handelsman RA, Lew JS (1970).

[16] Wong R (1974). Asymptotic Approximations of Integrals.

[17] Heinze J (1981). J Electroanal Chem.

